# Microstructure, Shrinkage, and Mechanical Properties of Concrete with Fibers and Experiments of Reinforced Concrete Beams without Shear Reinforcement

**DOI:** 10.3390/ma15165707

**Published:** 2022-08-18

**Authors:** Oldrich Sucharda, Zuzana Marcalikova, Radoslav Gandel

**Affiliations:** 1 Department of Building Materials and Diagnostics of Structures, Faculty of Civil Engineering, VSB-Technical University of Ostrava, Ludvíka Podéště 1875/17, 70800 Ostrava-Poruba, Czech Republic; 2 Department of Structures, Faculty of Civil Engineering, VSB-Technical University of Ostrava, Ludvíka Podéště 1875/17, 70800 Ostrava-Poruba, Czech Republic

**Keywords:** concrete, fiber-reinforced concrete, microstructure, mechanical characteristics, three-point bending test, reinforced concrete, beam

## Abstract

The current findings on concrete with fibers show that research has focused primarily on individual aspects, especially in terms of mechanical properties and structural uses. However, no broader view of the problems solved has been provided. In this study, we present a conceptual overview of a new, comprehensive experimental program for the assessment of fiber-reinforced concrete, which includes the analysis of microstructural and structural elements, as well as specific features such as shrinkage and resistance to pressurized water. The proposed experimental program included several variants of schemes for the dosing of fibers into concrete, using steel fibers that were short and straight. Fiber dosing was performed up to 110 kg/m^3^. The basic tests performed included tests of the compressive strength of concrete, and of the split and flexural tensile strength for different dosing amounts. Within the structural tests of reinforced concrete beams without shear reinforcement, two variants of spans with different degrees of reinforcement were implemented. Herein, the test results are evaluated graphically with a detailed analysis of the positive effect of fibers, and we also provide general recommendations for the structural uses of the fibers used and the design of fiber-reinforced concrete structures. Among the important results of this experimental program was the observation of a significant increase (of the order of tens) of the percentage of the split tensile strength and an increase of the overall load-bearing capacity of the reinforced concrete beams without shear reinforcement. Among the important aspects of our findings is the fact that a fine-grained concrete mixture was used, which increased resistance to pressure water seepage, and therefore, the effect of shrinkage can be influenced by the method of production and the treatment of the concrete used. We also provide detailed figures of the microstructure.

## 1. Introduction

As part of sustainable development, in technical areas, including construction, people strive to reduce costs or demands in the production of a particular product, while improving its useful properties. One of the options within concrete structures is fiber-reinforced concrete [1,2,3,4] or the addition of other materials into the recipe composition [5,6]. Fiber-reinforcement can significantly improve the mechanical tensile properties and fracture properties of concrete [7] and can contribute to the overall greater ductility and deformation capacities of structures. These positive aspects can significantly reduce the main disadvantages of plain concrete. However, the use of inverse analysis and suitable experiments is often necessary for the identification of fracture-mechanical parameters [7,8]. One of the interesting approaches to the verification of the mechanical properties of fiber-reinforced concrete is the use of small beams [9]. The possibility, in research tasks, of combining fibers [10] and the use of high-strength concrete [11] is also often related to the use of specialized tests [12], including abrasion resistance testing [13]. It is also important to deal with the problem of temperature and humidity effects [14].

Several recommendations, design codes, and standards are available for fiber-reinforced concrete, which provide basic information about this area of knowledge, for example, from the International Federation for Structural Concrete (FIB) [15,16], the International Union of Laboratories and Experts in Construction Materials, Systems and Structures (RILEM) [17,18], British Standards (BS) [19], and Deutscher Ausschuss für Stahlbeton (DAfStb) [20]. Fiber-reinforced concrete is itself the subject of extensive research [21,22,23], which, however, usually focuses only on a selected specific research focus. Typically, these are only microstructure, mechanical and physical properties, or design purposes. However, a comprehensive overview of research from microstructure to structural elements in fiber-reinforced concrete is often lacking, and the results cannot be unambiguously generalized with respect to its quasi-brittle nature and individual composition and dosage design. The results of experimental programs are also often not suitable for wider use, e.g., for numerical modeling, and they do not allow the use of inverse analysis, and, for example, do not include the testing of different sizes of structural elements.

**Figure 1:**
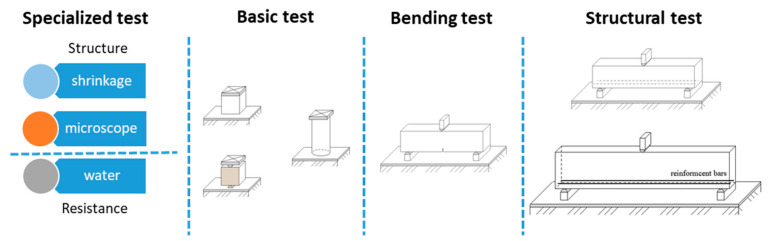
Concept of the experimental program used in this study.

The research concept presented here is shown in Figure 1. In this study, we focused on a specific experimental program, which addressed all the most important areas of materials engineering, and performed regression analysis for selected tests, in which the effect of fiber dosing was clarified.

**Figure 2:**
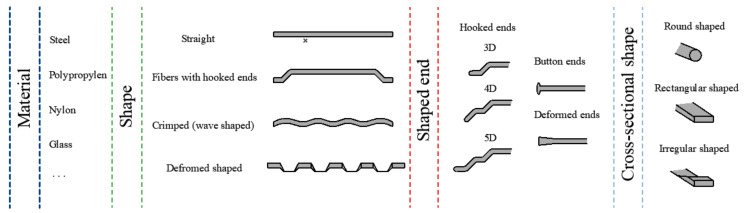
Types of fibers available for fiber-reinforced concrete.

The greater advantage—also related to its disadvantages—of fiber-reinforced concrete is that it allows the individual design of concrete compositions and the dosing of fibers, which makes it difficult to generalize knowledge and limits the possibility of structural design use. It should also be noted that there are many variants of fibers. Selected variants and possible divisions of fiber types for fiber-reinforced concrete are shown in Figure 2.

From the technological point of view regarding the production of concrete and fiber-reinforced concrete, it is necessary to ensure the homogeneity of a structure, and the resulting properties are also significantly affected by the concrete composition [24], especially in combination with high-performance concrete [25,26]. Among the typical demanding cases of fiber use is the case of high fiber dosing, above 75 kg/m^3^ [3], which makes processing and concreting of structures difficult [21,22,27,28,29]. Many mechanical properties and test procedures/standards are commonly included in the analysis of concrete and fiber-reinforced concrete [30,31,32,33]. Typically, these include compressive strength [30] and the modulus of elasticity [33]. However, the testing and identification of tensile properties is a specific area. It is often used to describe mechanical fractures [34,35,36,37], focusing on providing a generalized description of damage and crack propagation in quasi-brittle materials. In the case of compressive strength tests [30], test standard cubes and cylinders are most often used. In the assessment of tension mechanical properties, the number of types of test schemes is significantly larger. These can be divided into split tensile tests [32], axial tensile tests, and bending tests in three-point and four-point variants [31]. The use of fiber-reinforced concrete in the solution of construction problems is very wide and includes foundations, slabs on the ground [38,39,40], beams [41], industrial floors [42], and beams without shear reinforcement [43,44]. Importantly, the design and use of fiber-reinforced concrete needs to be approached qualitatively, because fibers affect basic mechanical properties and fracture properties [34,35], and the residual tensile strength and elastic properties of the material are particularly important [45]. Adequate knowledge of concrete and quasi-brittle materials should not only include information on compressive and tensile strength from the viewpoint of mechanical properties, but should also include details regarding crack initiation [46,47,48,49] and knowledge of the crack propagation process.

However, the current findings in the literature show that researchers and research tasks have focused primarily on individual aspects of fiber-reinforced concrete, especially in terms of its mechanical properties or structural uses. No broader view of the solved problem has been provided, and the partial results of individual studies cannot be easily connected based on previous research, considering the number of variants of fiber-reinforced concrete and the various individual composition designs presented. For example, Katzer [4] deals with quality and mechanical properties, Lantsoght [2] presents a database of tests of structural elements without shear reinforcement, Giaccio [9] deals with design parameters, and Abrishambaf [27] focuses on tensile strength. These studies only deal with partial aspects. They do not provide more detailed information about, for example, the concrete’s microstructure, shrinkage, or resistance to pressurized water.

The motivation for the present study was, therefore, to provide a comprehensive experimental program for the assessment of fiber-reinforced concrete, including the analysis of microstructural and structural elements, as well as specific features such as shrinkage and resistance to pressurized water.

## 2. Materials and Experimental Program

**Figure 3:**
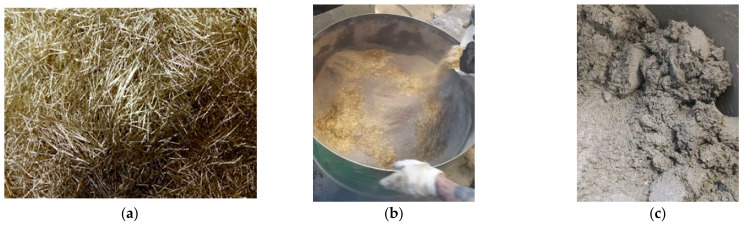
MasterFiber 482 fibers (**a**), dosing of the fibers in a mixer (**b**), and a concrete mix with fibers (**c**).

The research area of this study was focused on several goals, including basic mechanical properties, bending tests, structural tests, and specialized tests. Specialized tests included the study of the microstructure using optical and electron microscopy, shrinkage tests, and tests of resistance to pressurized water. A concrete matrix based on fine-grained concrete [12,44] was chosen for the experimental program. The maximum size of the aggregate was 4 mm. The fine-grained concrete was based on Portland cement, and further details are given in the manufacturer’s technical sheet [50]. The concrete met the ČSN EN 206 standard [51]. The formula was designed with the aim of obtaining increased resistance to deicing agents, pressurized water, and shrinkage. MasterFiber 482 (Master Builders Solutions) [52] fibers, as shown in Figure 3a, were chosen with regard to the availability of the production technology, with dosages ranging from 40 kg/m^3^ to 110 kg/m^3^ in up to 6 test series. The fiber parameters are presented in Table 1, and these were taken from the manufacturer [52]. The concrete mix production process is shown in Figure 3b and the finished mix is shown in Figure 3c.

## 3. Experimental Program of Basic Mechanical Properties and Results

**Figure 4:**
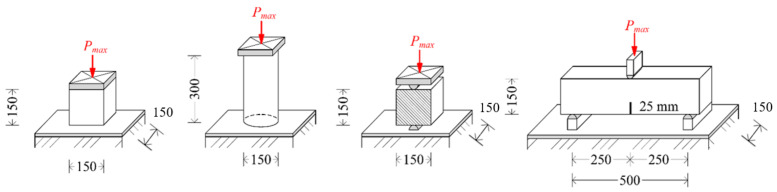
Test scheme of basic mechanical properties and bending test for concrete and fiber-reinforced concrete; cube 150 × 150 × 150 mm; cylinder 150 × 300 mm; beam 150 × 150 × 600 (700) mm, span 500 mm.

This section deals with the evaluation of the basic mechanical properties of fiber-reinforced concrete. The mechanical properties selected for study were the compressive strength, split tensile strength, and modulus of elasticity. A cube of nominal size 150 mm × 150 mm × 150 mm was chosen for compressive strength [30] and split tensile tests [32]. The modulus of elasticity was tested for cylindrical samples with only plain concrete for reference [33]. The test scheme used for the testing of basic mechanical properties and bending tests is shown in Figure 4.

Each series had three or six samples. The samples were tested after 28 days. The samples were stored in a water bath. Details on the number of samples and the test method are given in Table 2.

**Figure 5:**
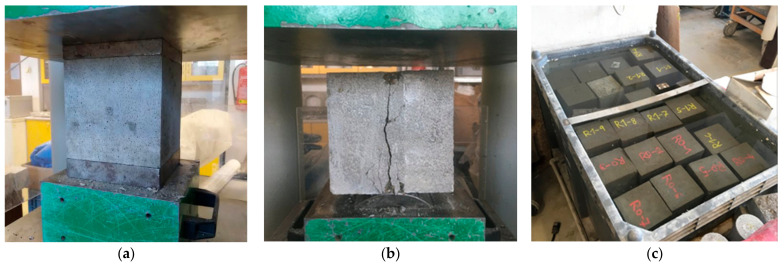
Test specimens before and after the test: (**a**) compressive strength test, (**b**) split tensile test, (**c**) storage of samples in water.

Sample photographs from the tests of basic mechanical properties are shown in Figure 5. The figure also shows the storage of test specimens in a water bath.

The results of the basic tests are summarized in Table 3 for the comparison of plain concrete and fiber-reinforced concrete, which was tested in five series with a dosage of 40 to 110 kg/m^3^. From the evaluation of the results of the bulk density testing, the effect of the dosing of fibers on the bulk density was clearly evident, gradually increasing from 2205 to 2303 kg/m^3^. The negative difference in the increase of bulk density was observed only for dosings of 90 and 110 kg/m^3^, and the difference was minimal. In the case of compressive strength, the positive effect of the fibers was evident.

However, the increase of strength was only observed for dosing amounts up to 75 kg/m^3^, and the compressive strength increased up to 64.01 MPa from the reference value of 57.1 MPa for plain concrete. However, for higher fiber dosing, the compressive strength began to decrease slightly, but still remained greater than the reference value of plain concrete. The decrease of compressive strength was probably also related to a decrease of bulk density, which results in an increase of porosity. High fiber dosing is characterized by significantly more difficult processing in concrete production.

The basic tests also included the determination of the static and dynamic moduli of elasticity, which were tested using cylinders. The reference value for plain concrete was determined with regard to the capacity of the laboratory and the small influence of fibers on the moduli. The resulting average static modulus of elasticity was 30.8 GPa, with a select variation coefficient of 3.6%, and dynamic modulus of elasticity 43.3 GPa, with a select variation coefficient of 1.6%.

## 4. Bending Test and Results

**Figure 6:**
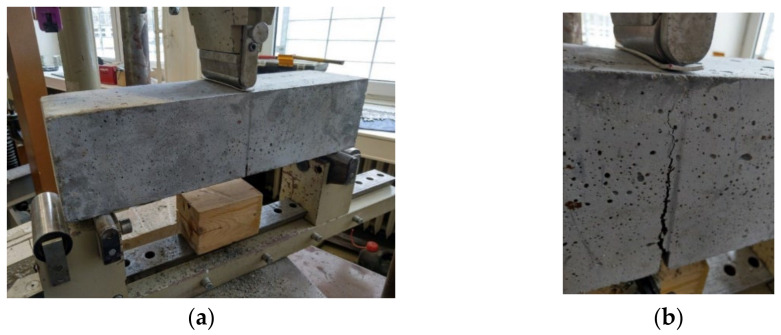
(**a**) Three-point bending test, (**b**) a crack in the concrete.

Another important type of tests are bending tests, which make it possible to determine the tensile strength in bending. Load-displacement diagrams are an important output of these tests. As part of the bending tests, three-point tests were performed with a notch of 25 mm and a span of 500 mm [12]. The results of the bending tests also include load-displacement diagrams. Details of the test series are presented, along with the numbers of samples, in Table 4. Figure 6a shows a three-point bending test with a notch and Figure 6b shows a crack in the test specimen.

**Figure 7:**
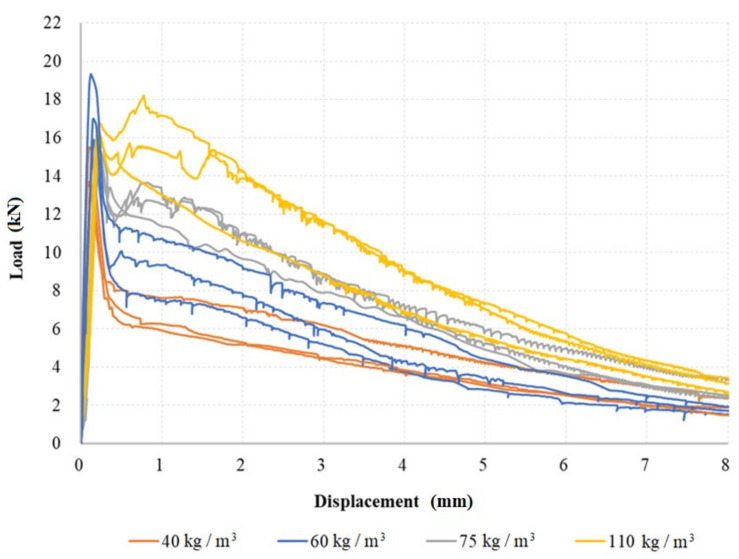
Load–displacement diagrams—three-point bending tests.

Bending tests of fiber-reinforced concrete were performed at fibers dosages of 40, 60, 75, and 110 kg/m^3^. Three samples (tests) were performed for each dose. The three-point bending test results are shown in Figure 7. In the load-displacement diagrams, one can observe that for all dosages, residual tensile strength was evident after the formation of a crack in the test beam. However, for dosages of 40 kg/m^3^, the residual strength was relatively small. For fiber dosages of 60 and 75 kg/m^3^, increases in the residual tensile strength were clearly visible. In the case of a fiber dosage of 110 kg/m^3^, the effect of tensile strengthening after the formation of the initial crack was noticeable. The final residual strength values observed for deformations of 8 mm were in a relatively small range for all tested specimens. Test samples with a fiber dosage of 60 kg/m^3^ show higher peak strength than the other samples, but the next section of the load-displacement curve displayed the expected residual tensile strength, between the curves of 40 and 75 kg/m^3^, as can be assumed. The higher peak load values observed for a fiber dosage of 60 kg/m^3^ could be caused by the material variability of fiber-reinforced concrete, and it would be appropriate to perform more tests. However, our overall assessment of the course of the curves has a representative character. The addition of fibers always increased the peak value of the load, and the influence of dosage was particularly visible on the residual tensile strengths, i.e., on the downward (softening) curve of the load diagram.

**Figure 8:**
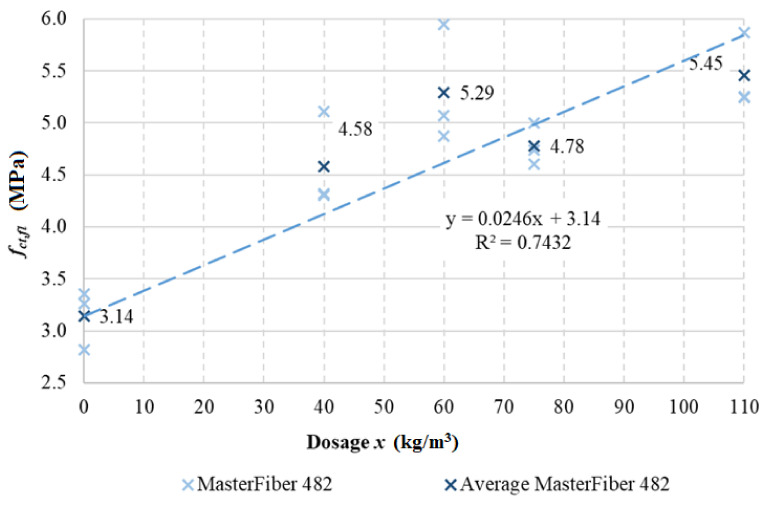
Bending tests of tensile strength.

For the results of the tensile tests, it was possible to determine the regression dependence of strength, depending on the dosing of the fibers. A graphical representation of these results is shown in Figure 8.

The influence of fibers on the mechanical properties of fiber-reinforced concrete was also analyzed by assessing functional dependence. In the case of split tensile strength, it is possible to formulate the resulting regressive analysis as follows:

*F_ct,sp_* = 0.0267*x* + 2.99, (1)

where *x* is the dosage of fibers (kg/m^3^), and the overall reliability is very high and reaches values greater than 0.996. In the case of bending tensile strength, the resulting regressive analysis is formulated as

*F_ct,fl_* = 0.0246*x* + 3.14, (2)

where *x* is the dosage of fibers (kg/m^3^), when the reliability is good and reaches a value of around 0.7432.

## 5. Perspective on the Microstructure of Concrete and Fiber-Reinforced Concrete

The study of the microstructure is one of the important sources of information on concrete materials. Particularly in the case of quasi-brittle materials, one can study the microstructure to obtain information about the character of the fracture surface and details about the bonds at the microstructural level. Two approaches were therefore chosen for this area, the first of which was focused on the fracture surface after destructive testing. The partial goal was to focus on inequalities for different components of the material composition of concrete. A digital optical microscope with an extremely large depth of field was used for the measurements, which also allowed the visualization of graphical images.

**Figure 9:**
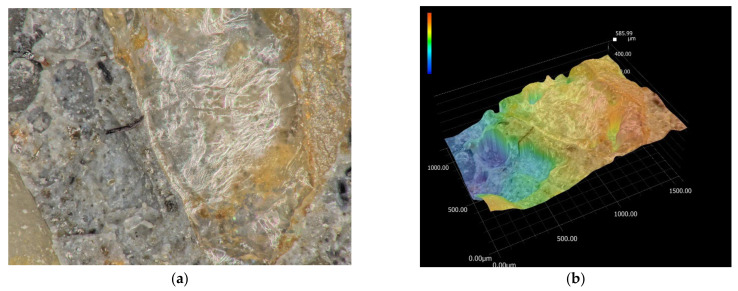
(**a**) Microstructure of 1000 × 1500 μm; (**b**) fracture surface of 1000 × 1500 μm: plain concrete (scale: bleu—min. 0; red—max. 585.99 μm).

**Figure 10:**
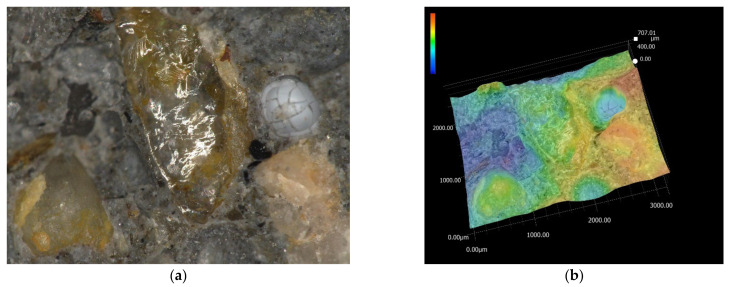
(**a**) Microstructure of 2000 × 3000 μm; (**b**) fracture surface of 2000 × 3000 μm: plain concrete (scale: bleu—min. 0; red—max. 707.01 μm).

**Figure 11:**
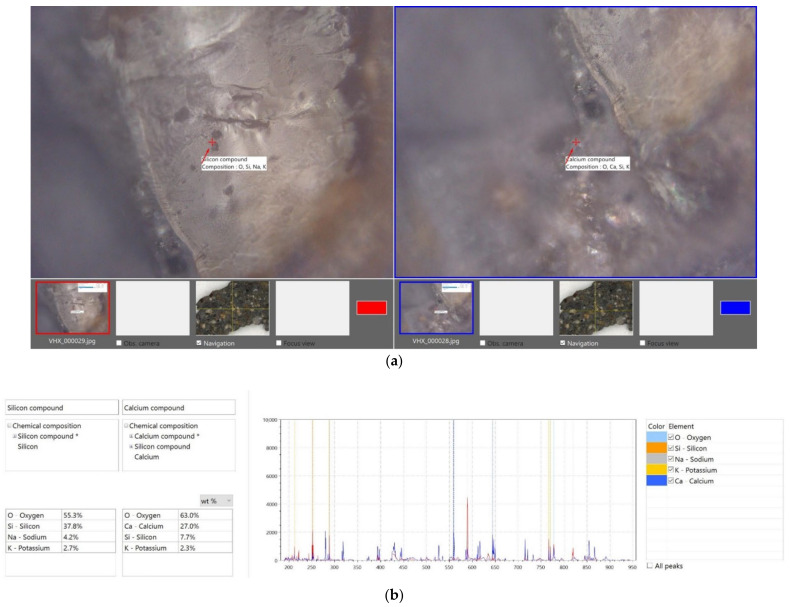
Material analysis using high-speed analysis (LIBS). (**a**) Microstructure photograph, (**b**) elemental composition for the selected location of the microstructure.

The first selected area is shown in Figure 9a and covers an area of 1000 × 1500 μm. The different material components of the structure of fiber-reinforced concrete are also clearly visible in Figure 9. For a better study of the structure, it is possible to visualize the surface, where the maximum difference in the surface is 585.99 μm. In further research, scanning of a two-fold area was performed, as shown in Figure 10, which covers an area of 2000 × 3000 μm. In this case, the differences in the concrete components were even more pronounced, and the maximum difference in the surfaces reached 707.01 μm. These analyses were followed by material analysis using high-speed analysis LIBS (Laser-Induced Breakdown Spectroscopy), which is one of the methods of atomic emission spectroscopy. Two different sites from the laboratory sample were chosen for the analysis of the elements, and the sites are marked in Figure 11. The results of this analysis revealed that the main element content was oxygen for both sites, at around 55%–63%. In the case of the first site, silicon (37%) and sodium (4%) also had a significant share. The composition differed for the second site, with a more significant proportion of calcium (27%) and silicon (7%). The results of the analysis were in agreement with the expected assumptions. The determined composition corresponded to our assumptions, as we expected to observe the required mechanical properties and resistance.

**Figure 12:**
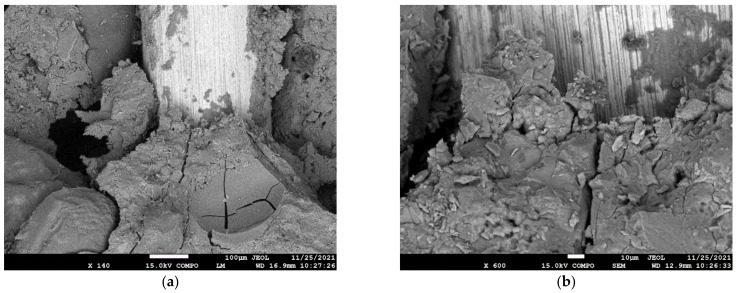
Microstructure (**a**) scale 140× (**b**) scale 600×—fiber-reinforced concrete (90 kg/m^3^).

**Figure 13:**
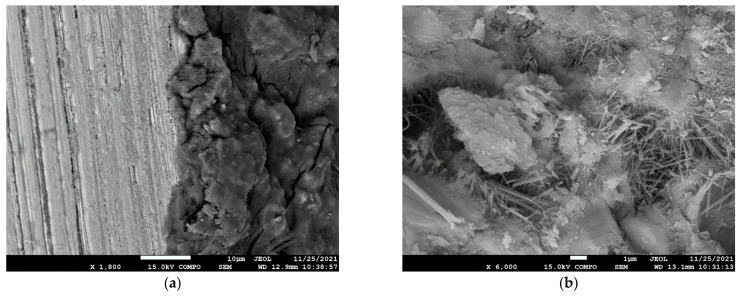
Microstructure (**a**) scale 1800× (**b**) scale 6000×— fiber-reinforced concrete (90 kg/m^3^).

Another part of the study of the microstructure focused on a more detailed depiction of the connection between the concrete and the steel fibers. An SEM (Scanning Electron Microscope), which is an electron microscope that uses a moving electron beam for imaging, was used for this investigation. The specific area between the concrete matrix and the fiber, with a magnification 140×, is shown in Figure 12a. A more detailed magnification of 600× is shown in Figure 12b, and a magnification of 600× is shown in Figure 13a. The electron microscope made it possible to further focus on details of the structure for a magnification of 6000×. The individual parts of the microstructure are shown in Figure 13b. The main advantage of the chosen fine-grained concrete was its dense microstructure, which is a prerequisite for high strength and high resistance. The dense microstructure was the result of an appropriate water-to-binder ratio and adherence to production technology. This was confirmed by the images taken of the microstructure. Figure 12 and Figure 13 clearly show the product of the hydration process and the resulting microstructures, which created a strong structure between the concrete components (aggregate) and the steel fibers.

## 6. Depth of Penetration with Pressurized Water

**Figure 14:**
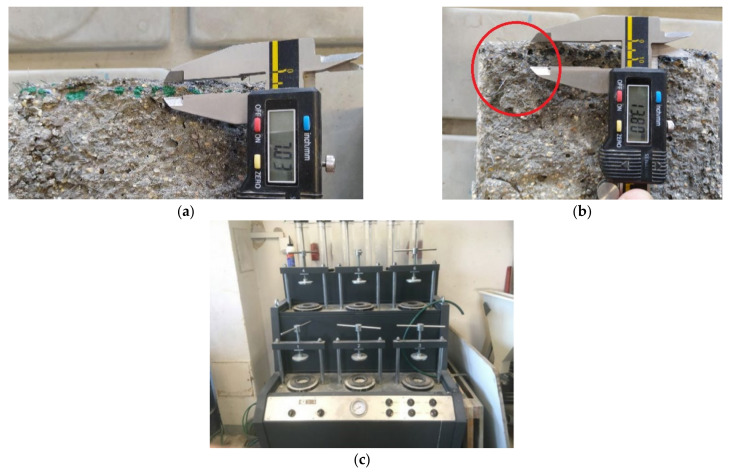
Seep depth experimental samples. (**a**) Fiber-reinforced concrete—sample 1; (**b**) fiber-reinforced concrete—sample 2; (**c**) machine for testing the depth of penetration with pressurized water in concrete.

Another part of this study focused on finding the effect of fibers on the pressurized water resistance [53] of concrete, using a testing machine depicted in Figure 14c. Through tests of basic mechanical properties and with adherence to the complexity of sample production, it was also found that dosing above 90 kg/m^3^ was already technologically inefficient and uneconomical. With regard to the limited number of samples and finding the largest possible difference, a dosage of 90 kg/m^3^ was chosen for the specialized test. The concrete matrix without fibers that was designed could be typically used for repairs or for the water management of structures with increased durability. The depth of penetration with pressurized water was tested on three cube samples made of plain concrete and three fiber-reinforced concrete cube samples with a fiber dosage of 90 kg/m^3^ and an edge length of 150 mm.

Table 5 shows the pressurized water penetration values of the individual samples and their averages. Based on the measured depths of penetration with pressurized water, it follows that the presence of fibers in the concrete mix could adversely contribute to its watertightness, which may result in a reduction in its durability. Figure 14 shows the measurement of the penetration depth with pressurized water on a fiber-reinforced concrete sample. The leakage depth shown in Figure 14 reached a higher value locally on this sample, namely, 13.8 mm, which was caused by a clump of fibers near the surface of the sample (see the area marked in Figure 14). However, according to a study of the surface and structure, it can be stated that the increased depth of leakage would probably be eliminated in a more suitable way through the final surface treatment.

## 7. Volume Changes—Shrinkage

**Figure 15:**
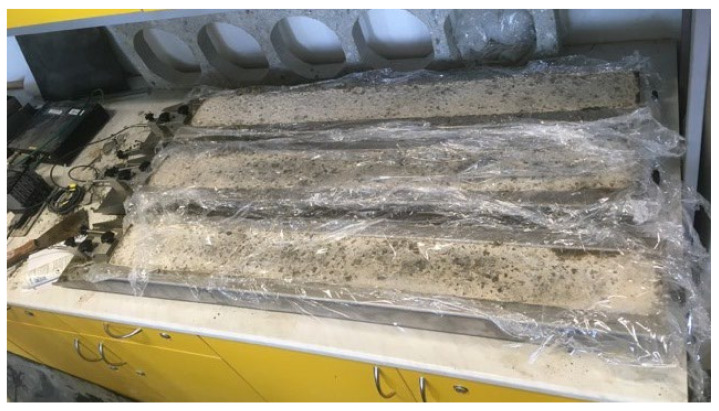
Test equipment during the measurement of volume changes in measuring troughs.

The monitoring of volume changes with *Schleibinger Testing Systems* [54] took place in two phases. In the first phase, from 16 December 2020 for a period of 42 days, volume changes in plain concrete were recorded. In the second phase, from 3 March 2021 for a period of 42 days, volume changes in the fiber-reinforced concrete mixture were recorded, using MasterFiber 482 steel fibers with a dosage of 90 kg/m^3^. In both cases, volume changes were observed in three mixtures in measuring troughs measuring 60 × 100 × 1000 mm (with a height of 60 mm) for 42 days, with the mixtures in the two troughs being wrapped in a protective foil throughout the maturation process, and a mixture in one gutter matured in the air (see Figure 15). Samples wrapped in foil were used to simulate ideal conditions with regard to concrete treatment. For a sample, when there is air access, the sample may be more susceptible to drying and this is typical for cases of bad technological discipline.

The objective of this test was to analyze the effect of fibers on the deformations caused by shrinkage and expansion of the volume.

**Figure 16:**
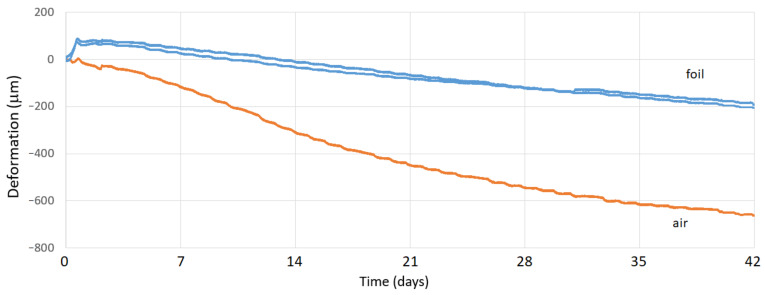
Volume changes in plain concrete over 42 days.

**Figure 17:**
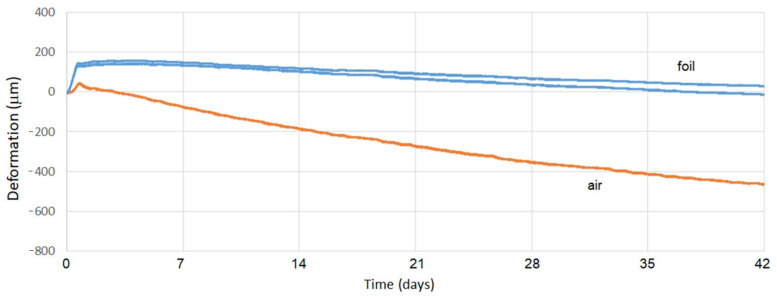
Volume changes of fiber-reinforced concrete over 42 days.

It is clear from the graphs in Figure 16 and Figure 17 that the protective film significantly reduced the overall volume changes in both mixtures. In the initial phase, the gutters in which the surface of the monitored mixtures was covered with foil showed a more significant expansion than in the mixtures of uncoated foils. However, the increase of volume was relatively small, relative to the total final deformations. After 42 days, the volume changes of plain concrete showed greater changes compared to fiber-reinforced concrete.

In both cases, the mixtures without protective foil (with air contact) troughs showed a smaller increase of extensibility in the first days, but at the end of the test period, the shrinkage values were incomparably higher than those for the mixtures in the gutters provided with the protective foil. It was also true that the fiber-reinforced concrete samples achieved lower shrinkage values compared to the plain concrete samples. In Table 6, we present the final volume changes of both mixtures.

An evaluation of the results presented in Table 6 (where negative values represent shrinkage and positive values represent volume expansion) shows that the presence of fibers in the concrete mix has a positive effect on its volume changes. In all cases, the volume changes of fiber-reinforced concrete were smaller than the original state in plain concrete. The highest shrinkage for fiber-reinforced concrete was 466 µm, whereas for plain concrete, it was 663 µm. It is also clear from the table that the samples of both mixtures covered with foil achieved smaller values of volume changes overall. The treatment of the composites at the time of maturation is therefore an important task which contributes significantly to reducing their volume changes.

## 8. Reinforced Concrete Beams without Shear Reinforcement—Structural Testing

Applications of fiber-reinforced concrete can be found, for example, in reinforced concrete beams without shear reinforcement, where its use can be very advantageous from a technological point of view [44]. For this reason, we focused on this area, and two types of beams were chosen for the span of 600 (small beams) and 900 mm (beams) with different cross-sections. In both cases, the beams were reinforced with B500 concrete reinforcement, with a diameter of 10 mm. Each beam had two bars, and the reinforcement cover was 20 mm. The beams were tested for three-point bending and the load-bearing capacity was determined. The nominal size of the smaller beam was 150 × 150 × 700 mm. The tests were performed for dosages of 40, 60, 75, and 90 kg/m^3^.

**Figure 18:**
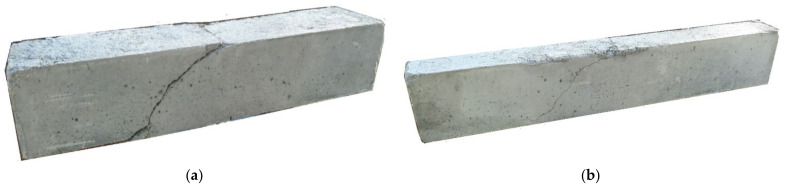
Reinforced concrete beam without shear reinforcement after the test (**a**) span: 600 mm; (**b**) span: 900 mm.

**Figure 19:**
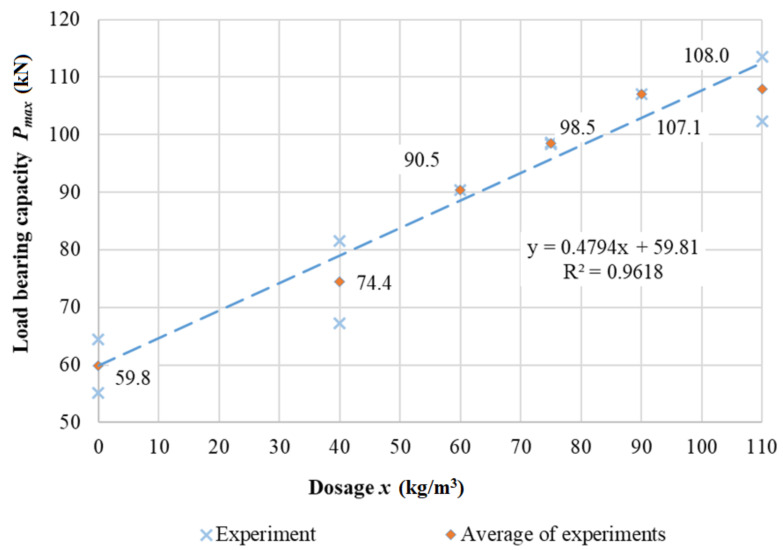
Load-bearing capacity—reinforced concrete beam without shear reinforcement. Span: 600 mm.

A selected image of a small beam specimen after the experiment is shown in Figure 18a, where a shear crack is clearly visible. The experimental results of smaller reinforced concrete beams without shear reinforcement are shown in Figure 19. According to our assumptions, the maximum load capacity during beam tests with a dosage of 110 kg/m^3^ was. The average maximum load bearing capacity was 108.0 kN for the beams with dosage.

A regression analysis was performed, and the agreement reached R^2^ = 96.18%. Thus, we can assume a very high dependence of the maximum load-bearing capacity on the dosing of MasterFiber 482 [52] fibers.

The function of the dependence of the achieved load-bearing capacity, *P_max_*_,600_, in the testing machine on the dosing of fibers in the three-point bending test for small beams has the following form:

*P_max_*_,600_ (kN) = 0.4794*x* + 59.81, (3)

where *x* is the dosing of fibers (kg/m^3^). The second variant of the bending test was for beams with nominal dimensions of 190 × 100 × 1150 mm (height 190 mm) and a span of 900 mm. As in the previous test, B500B reinforced bars with a diameter of 10 mm, two pieces in number, and a reinforcement cover of 20 mm were chosen. With regard to the dimensions and capacity of the laboratory, tests were performed for fiber dosages of 60 kg/m^3^ and 90 kg/m^3^.

**Figure 20:**
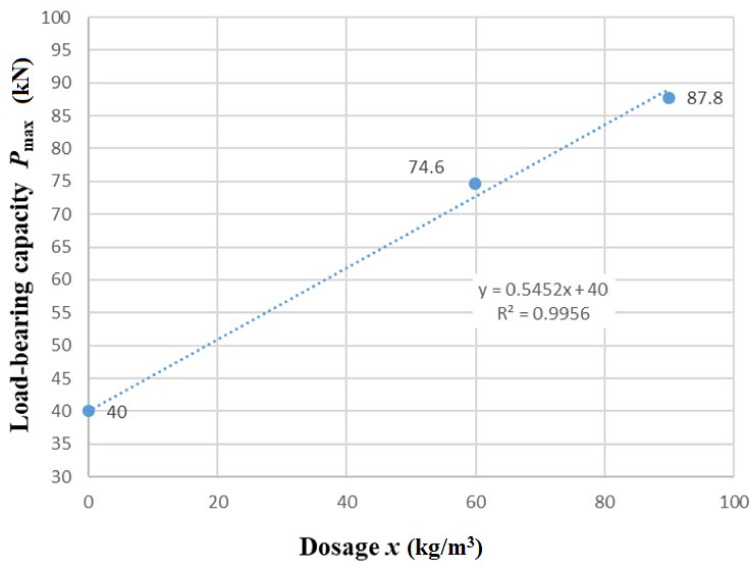
Load-bearing capacity—reinforced concrete beam without shear reinforcement. Span: 900 mm.

The mechanism of failure was very similar in both cases. A shear crack formed in the beam. A selected beam after testing is shown in Figure 18b. The resulting total loads are shown graphically in Figure 20.

The function of the dependence of the achieved load-bearing capacity, *P_max_*_,900_, in the testing machine on the dosing of fibers in the three-point bending test for beams has the following form:

*P_max_*_,900_ (kN) = 0.5452*x* + 40.0, (4)

where *x* is the dosing of fibers (kg/m^3^). The results of the graph in Figure 20 show that the load-bearing capacity of a fiber-reinforced concrete beam with concrete reinforcement and a fiber dosage of 90 kg/m^3^ was approximately 13 kN higher than that of a beam with a dosage of 60 kg/m^3^, which represents an increase of load-bearing capacity of approximately 17.5%. Based on the results of both bending tests, it can be concluded that the presence of steel fibers in the reinforced concrete structural elements contributes favorably to its load-bearing capacity.

## 9. Discussion

This study deals with the field of fiber-reinforced concrete. The introduction contains information about fiber-reinforced concrete, including the most important testing and research methods. The main motivation for this research was to provide a comprehensive view of the research area of fiber-reinforced concrete, ranging from its microstructure to its use in structural elements. The basic part of the manuscript was focused on determining the mechanical properties of fiber-reinforced concrete and monitoring the effect of fiber dosing on these properties, including regression analysis. The tests included a plain concrete mix as a reference. Based on the compressive strength results, it can be stated that with an increasing proportion of fibers in the fiber concrete mixture, the compressive strength also increases, but suitable processing of the concrete mixture must be ensured.

The split tensile strength increased from 2.99 to 5.88 MPa, i.e., almost twofold. The increase of split tensile strength was significantly pronounced already for the dosage of 40 kg/m^3^ up to a value of 4.18 MPa. The very positive effect of the fibers was documented by three-point bending tests, and residual tensile strength after peak loading was evident.

The bulk density of the concrete sample must also increase. However, a steady increase of bulk density was not achieved for all dosages. With regard to the primary use of steel fibers in concrete, the results of split tensile and bending strength tests are more relevant.

One of the important parts of this study was the study of the microstructure, which verified that the fracture surface showed relatively small differences. In addition, elemental composition analysis was also performed for selected areas. We have provided detailed images of the matrix between the concrete and the steel fiber. The results of the analysis were in agreement with the expected assumptions, displaying the expected mechanical properties and resistance.

Among the important results of this experimental program, we verified the effect of the use of fibers on volume changes in concrete with respect to different environments (foil/air). The fibers had a significant positive effect on the elimination of volume changes. Based on the measured depths of penetration with pressurized water, we found that the presence of fibers in the concrete mix can adversely contribute to its watertightness, which may result in a reduction in its durability. However, the resulting water penetration values were minimal and a high resistance of concrete against pressurized water was observed. Through implementing similar studies of the surface and structure, it can be stated that the increased depth of leakage could probably be eliminated in a more suitable way through a final surface treatment.

After the basic tests of mechanical properties, dosages of 60 and 90 kg/m^3^ were selected for the testing of structural elements. The structural elements were reinforced concrete beams without shear reinforcement, which can present a typical suitable use of fiber-reinforced concrete. Based on the results, it can be stated that increasing the content of steel fibers from 60 to 90 kg/m^3^ increased the load-bearing capacity by approximately 17.5%. However, in comparison with reinforced concrete beams made of plain concrete, the overall load-bearing capacity was increased by almost twofold.

In the case of dosing steel fibers above 90 kg/m^3^, it is necessary to realize that the workability of the concrete mixture deteriorates significantly. It is also important to monitor the even distribution of the fibers very carefully. The samples can also have a more problematic surface treatment, which greatly influences the results regarding the resistance to penetration with pressurized water. There can also be problems with the compaction of the mixture itself.

## 10. Conclusions

The research program implemented here included experiments and results assessing both microstructural and structural elements, as well as specific tests on features such as shrinkage and resistance to pressurized water. The results and information obtained through the research program can be described in the following partial conclusions.

- The positive effect of fibers on the tensile strength of fine-grained concrete with aggregates up to 4 mm was verified. With higher dosages of fibers, the tensile strength was also greater, both in the split tensile tests and in the bending tests. The deformation capacity of concrete was increased significantly.
- In the bending test, the effect of higher dosing on the post-peak course of the load-displacement diagram was clearly visible.
- For the practical application and dosing of fibers in concrete, the production technology is limiting, and dosages above 90 kg/m^3^ are very difficult to process.
- Typically, higher fiber dosing can adversely affect compressive strength, which can also result in a change in the microstructure, a pore volume increase, or a slight increase or decrease of bulk density.
- The advantages of using fiber-reinforced concrete include the fact that it contributes to reducing the impact of the shrinkage of concrete, as has been experimentally verified.
- The results of the microstructure analysis were in agreement with the expected assumptions regarding structural bonds. The analyzed composition corresponded to the assumptions, exhibiting the expected mechanical properties and resistance. The results of the experimental program provide a new perspective on the detailed microstructure of concrete and fiber-reinforced concrete.
- In the case of the requirement of resistance to pressurized water in fiber-reinforced concrete, it is necessary to very carefully check the processing technology and the final surface treatment used. The use of fibers in concrete can affect the resistance to pressurized water locally.
- The very positive effect of the use of fibers was verified by experiments of reinforced concrete beams without shear reinforcement, with tests performed for different spans and cross-sections. The experiments reported here are suitable for numerical modeling, and the authors will focus on this area in further research.

## Figures and Tables

**Table 1:** Parameters of MasterFiber 482 fibers.

Parameters	MasterFiber 482	Unit
Fiber shape	Straight	
Bundling	Loosely	
Length *l*	13	mm
Diameter *d*	0.20	mm
Aspect ratio	65	(*l*/*d*)
Tensile strength	2200	MPa
Modulus of elasticity	200	GPa

**Table 2:** Parameters of basic tests and the number of samples (number).

Test of Parameters	Plain Concrete	Fiber Dosage (kg/m^3^)				
		40	60	75	90	110
Compressive strength [30]	6	6	3	6	3	6
Split tensile strength [32]	6	6	3	6	3	6
Modulus of elasticity [33]	3	-	-	-	-	-

**Table 3:** Results of the testing of basic mechanical properties.

Concrete	Dosage (kg/m^3^)	Bulk Density (kg/m^3^)	Standard Deviation of Bulk Density (kg/m^3^)	Compressive Strength—Cube (MPa)	Standard Deviation of Compressive Strength (MPa)	Split Tensile Strength—Cube (MPa)	Standard Deviation of Split Tensile Strength (MPa)
**Plain** **concrete**	0	2205	49.13	55.87	1.02	2.99	0.37
**FRC–MasterFiber 482**	40	2248	19.26	57.10	3.53	4.18	0.27
	60	2254	53.91	58.41	1.61	4.62	0.08
	75	2273	25.59	64.01	2.73	5.01	0.55
	90	2303	52.33	61.11	2.36	5.34	0.36
	110	2294	23.39	59.32	2.44	5.88	0.35

**Table 4:** Parameters of bending tests and numbers of samples (number).

Test of Parameters	Plain Concrete	Fiber Dosage (kg/m^3^)			
		40	60	75	110
Three-point bending test [31]	6	3	3	3	3

**Table 5:** Depth of penetration with pressurized water (mm).

Seep Depth	Fiber-Reinforced Concrete	Plain Concrete
Measurement–sample 1	7.03	6.80
Measurement–sample 2	13.80	7.27
Measurement–sample 3	6.88	5.73
Average	9.24	6.60

**Table 6:** Total volume changes—shrinkage. Time: 42 days.

	Fiber-Reinforced Concrete	Plain Concrete
Foil	yes/yes/no	yes/yes/no
Sample	F1/F2/F3	C1/C2/C3
(µm)	−13/31/−466	−205/−189/−663

## Data Availability

Data are contained within the article.
